# Children and adults with Attention-Deficit/Hyperactivity Disorder cannot move to the beat

**DOI:** 10.1038/s41598-017-11295-w

**Published:** 2017-09-14

**Authors:** Frédéric Puyjarinet, Valentin Bégel, Régis Lopez, Delphine Dellacherie, Simone Dalla Bella

**Affiliations:** 1 EuroMov Laboratory, University of Montpellier, 700 Av. du Pic Saint Loup, 34090 Montpellier, France; 2 NaturalPad, SAS, 700 Av. du Pic Saint Loup, 34090 Montpellier, France; 3 National reference center for narcolepsy and idiopathic hypersomnia, specialized in adult ADHD, Gui-de-Chauliac University Hospital, 80 Av. Augustin Fliche, 34295 Montpellier, France; 4 Inserm Unit U1061, La Colombière University Hospital, 39 Av. Charles Flahault, 34093 Montpellier, France; 5 Department of Psychology, PSITEC-EA 4072 Laboratory, University of Lille, Domaine Universitaire Pont de bois, 59653 Villeneuve d’Ascq, France; 6 University Hospital of Lille, Department of Pediatric Neurology, 2 Av. Oscar Lambret, 59037 Lille, France; 7 International Laboratory for Brain, Music and Sound Research (BRAMS), 1430 Boulevard du Mont-Royal, Montreal, QC H2V 2J2 Canada; 8 Institut Universitaire de France, 1 Rue Descartes, 75231 Paris, France; 9 Department of Cognitive Psychology, WSFiZ in Warsaw, Ul. Pawia 55, 01-030 Warsaw, Poland

## Abstract

Children and adults with Attention-Deficit Hyperactivity Disorder (ADHD) fail in simple tasks like telling whether two sounds have different durations, or in reproducing single durations. The deficit is linked to poor reading, attention, and language skills. Here we demonstrate that these timing distortions emerge also when tracking the beat of rhythmic sounds in perceptual and sensorimotor tasks. This contrasts with the common observation that durations are better perceived and produced when embedded in rhythmic stimuli. Children and adults with ADHD struggled when moving to the beat of rhythmic sounds, and when detecting deviations from the beat. Our findings point to failure in generating an internal beat in ADHD while listening to rhythmic sounds, a function typically associated with the basal ganglia. Rhythm-based interventions aimed at reinstating or compensating this malfunctioning circuitry may be particularly valuable in ADHD, as already shown for other neurodevelopmental disorders, such as dyslexia and Specific Language Impairment.

## Introduction

More than 5% of children show poor concentration, impulsivity and visible signs of hyperactivity^1^, either alone or in combination. This condition, named Attention Deficit/Hyperactivity Disorder (ADHD), is the most common neurobehavioral disorder of childhood^2^. Akin to other neurodevelopmental disorders such as dyslexia and Developmental Coordination Disorder^1^, it comes with poor school success and socioeconomic disadvantages^3^. While many children outgrow it, in about 50% of the cases ADHD carries over into adulthood with negative consequences at work and in everyday life^4^.

Children and adults with ADHD also struggle in perceiving and reproducing event durations. They have difficulties in telling or reproducing the duration of visual and auditory stimuli and in comparing time intervals^5^. Distortions in duration perception and production are also reported in other neurodevelopmental disorders such as autism spectrum disorders and dyslexia^6, 7^. Impaired timing is associated with poor reading, attention and language skills, and with impaired executive functions^5, 8^. The neural substrates of processing event durations (i.e., *duration-based timing*) include cerebellar-cortical pathways^9^. Structural anomalies in these brain regions (e.g., inferior or posterior vermis) and impaired connectivity within fronto-cerebellar networks are found in ADHD^10^. Thus, it does not come as a surprise that the processing of event duration is impaired in ADHD.

Owing to these difficulties in encoding and producing single durations, one may conclude that children with ADHD have a poor appraisal of the timing of events altogether. This conclusion may be premature, though. Children with ADHD might still be able to treat durations when embedded in a rhythmic context, by benefitting from its predictable temporal structure (i.e., by tracking the beat). Typically, durations can be processed more easily by the healthy brain when they form a rhythmic structure^11, 12^. The mechanism underlying beat tracking is based on relative analysis of time intervals, and referred to as *beat-based timing* ^9, 12–15^. Beat tracking is involved in both perceptual and motor tasks, such as detecting a subtle deviation from a beat (e.g., in music), or moving to the beat. This ability can be selectively spared following brain damage (e.g., spinocerebellar ataxia) in the presence of impaired processing of single durations^16^. Beat-based timing has been linked to partly independent neuronal networks, engaging basal-ganglia-cortical circuitries^12, 14, 16, 17^.

To our knowledge, the possibility that children with ADHD may have difficulties in rhythm perception has been suggested by one previous study^18^; children with ADHD revealed poorer performance than controls in detecting a change in duration in a rhythmic sequence. However, it is still unknown whether children with ADHD are generally impaired in beat perception in a wider array of tasks (perceptual and sensorimotor), and whether they can take advantage of a rich rhythmic stimulus (e.g., music) as compared to a simpler one (i.e., a metronome). This possibility is particularly appealing, as rhythmic auditory stimulation may serve as a remediation strategy in ADHD, as done for other timing disorders, such as Parkinson’s disease^19^, which displays poor beat tracking skills^15^. Interestingly, ADHD has been consistently associated to structural anomalies of the basal ganglia and malfunctioning basal-ganglia-cortical connectivity both in children^20^ and in adults^21^. For example, structural asymmetries in the caudate^22^, and lower activity in the putamen^23^ are reported in ADHD. The caudate is known to be preferentially linked to cognitive functioning, while the putamen is more closely related to sensorimotor functions^24^.

Due to the aforementioned malfunctioning of basal ganglia-cortical circuitries in ADHD, it is expected that children with this condition may have difficulties to track the beat, on top of their poor appraisal of single durations. To test this hypothesis, children with ADHD were asked to track the beat of rhythmic sound sequences. Metronomes and music were used to test the ability to both track a periodic sound and to internally generate a beat (based on music), a function typically associated with the activity of the basal ganglia^12, 25^. Children were asked to detect subtle deviations from the beat of music and metronome sequences. Moreover, they tapped with their finger to the beat of the same stimuli. For comparison, their ability to perceive single durations (duration-based timing) was tested by asking children to compare the duration of pairs of tones.

In addition, half of the tested children in the present study showed Developmental Coordination Disorder (DCD)^1^ on top of ADHD. DCD is characterized by impairments in activities that require motor coordination, and co-occurs with ADHD in about 50% of the cases^26–30^. There is evidence that children with DCD, like children with ADHD, are more variable in rhythmic motor tasks such as tapping than healthy controls^5, 31^. Increased abnormalities in motor network connectivity in children with both DCD and ADHD compared to ADHD alone have been recently described^32^. This would negatively impinge on beat tracking in motor tasks. However, DCD is not expected to affect beat tracking in purely perceptual tasks more than ADHD does. Finally, to assess whether the same pattern of timing disorders carries over into adulthood, a group of adults with ADHD was tested with a subset of the same beat tracking tasks.

## Results

### Duration and beat perception

**Figure 1:**
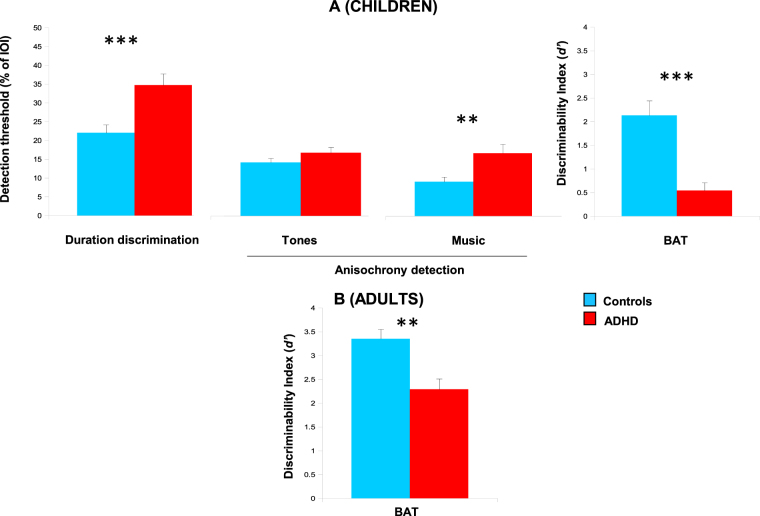
Beat tracking skills measured with perceptual tasks of BAASTA for children and adults with ADHD, and healthy controls. (**A**) Perception of duration tested in children with ADHD and controls using the Duration discrimination task, and beat tracking skills assessed with Anisochrony detection tasks (with tones and music) and the Beat Alignment Test (BAT). (**B**) Performance of adults with ADHD and controls tested with the BAT. Error bars are SEM. ***P < 0.0001, **P < 0.01.

Children with ADHD showed general difficulties in perceiving durations and in tracking the beat, relative to a group of matched controls (see Fig. 1A, for the results in duration discrimination, and beat perception tasks). Because children with ADHD, with and without DCD, did not differ on these tasks, we report only data from the pooled group.

Summary statistics for the two groups are presented in Table S1 (*SI Differences between ADHD and ADHD-DCD children performance*). Children with ADHD performed worse than healthy controls when asked to discriminate single durations [*t*_(37.8)_ = 4.13, *P* < 0.0001, *d* = 1.31]. This difference disappeared when children detected a deviant duration in the context of a periodic sequence of tones (Anisochrony detection with tones). However, poor beat tracking in children with ADHD relative to controls was apparent with music (Anisochrony detection with music) [Group x Stimulus interaction; *F*_(1,38)_ = 4.91, *P* < 0.05, *η* ^2^_*partial*_ = 0.11; group difference with tones, *t*_(36.3)_ = 1.69, *P* = 0.10, *d* = 0.53; with music, *t*_(38)_ = 3.58, *P* < 0.001, *d* = 1.19]. Finally, children with ADHD had difficulties in judging whether a sound was misaligned or not with a musical beat, as compared to controls [BAT; *t*_(17.6)_ = 4.70, *P* < 0.0001, *d* = 1.48]. This group difference persisted when covarying for duration discrimination performance, hence confirming a genuine impairment in beat tracking irrespective of poor coding of single durations [ANCOVA, *F*_(1,41)_ = 25.99, *P* < 0.00001, *η* ^2^_*partial*_ = 0.39].

In sum, children with ADHD could detect a deviation from the beat for a simple periodic auditory signal. Yet, they failed to do so with music. This is compelling evidence in favor of a beat-based deficit in ADHD. Beat tracking in music requires extracting periodicities at different embedded time scales from a complex auditory signal. Interestingly, poor beat tracking persisted into adulthood (see Fig. 1B). Adults with ADHD were poorer than age-matched controls in detecting whether a sound and a musical beat were aligned or not [BAT; *t*_(37)_ = 3.50, *P* < 0.001, *d* = 1.20]. Overall, adults outperformed children on this task [main effect of Age, *F*_(1,85)_ = 51.11, *P* < 0.00001, *η* ^2^_*partial*_ = 0.38], and all participants, regardless of age and group, showed worst beat tracking performance when music was presented at a fast tempo than at a slow tempo [main effect of Tempo, *F*_(2,170)_ = 7.41, *P* < 0.001, *η* ^2^_*partial*_ = 0.07; for details, see Fig. S1]. No interaction was found between Age group and presence/absence of ADHD.

### Tapping to the beat

**Figure 2:**
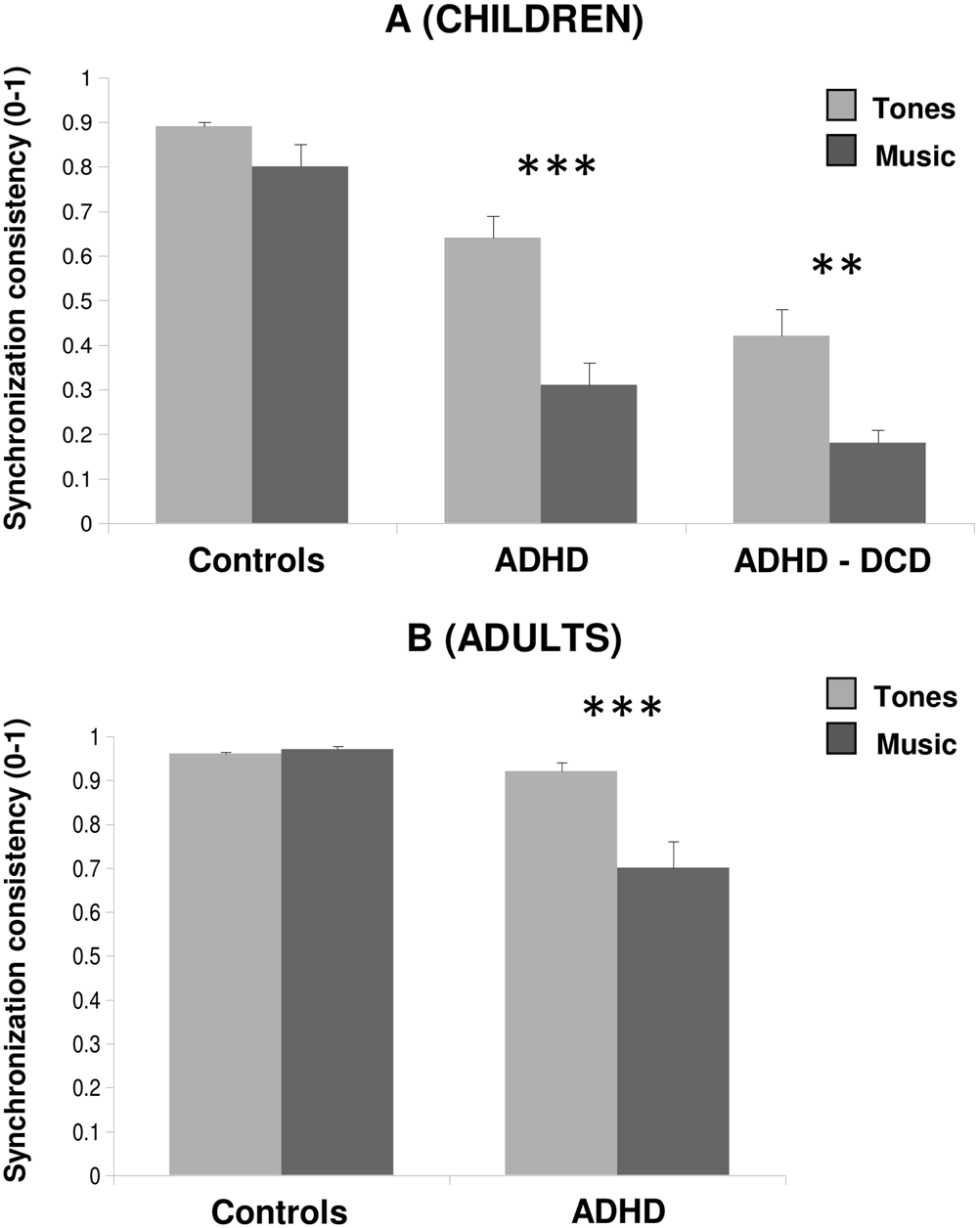
Beat tracking skills measured with motor tasks (finger tapping) of BAASTA for children with ADHD/ADHD-DCD, adults with ADHD, and healthy controls. (**A**) Performance of children with ADHD and controls when they tapped their finger to the beat of tone sequences and to music. Performance is expressed by the consistency of synchronization, from 0 to 1. Greater values indicate better synchronization to the beat. (**B**) Performance of adults with ADHD and controls on the same tapping tasks. Error bars are SEM. ***P < 0.0001, **P < 0.01.

Poor beat tracking in children with ADHD was also apparent when they tapped their finger to the beat of a metronome and of music (Fig. 2A). In this motor task, a difference was apparent between children with and without DCD. Children with ADHD and DCD exhibited worse synchronization to the beat (i.e., lower synchronization consistency) than children with only ADHD, and even worse than age-matched controls [main effect of Group, *F*_(2,51)_ = 37.61, *P* < 0.00001, *η* ^2^_*partial*_ = 0.60; ADHD-DCD < ADHD, *t*_(38.8)_ = 3.07, *P* < 0.01, *d* = 0.96]. Moreover, these group differences varied with stimulus complexity [Group x Stimulus interaction, *F*_(2,51)_ = 4.20, *P* < 0.05, *η* ^2^_*partial*_ = 0.14]. While controls tapped similarly well to both tones and music [*t*_(13)_ = 2.37, *P* = 0.10, *d* = 0.38], children with ADHD showed more difficulty when tapping to the musical beat than to tones [with DCD, *t*_(18)_ = 4.25, *P* < 0.01, *d* = 0.95; without DCD, *t*_(20)_ = 6.17, *P* < 0.0001, *d* = 1.33]. Notably, these findings were replicated across different tempos (see Fig. S2).

Adults with ADHD showed a similar pattern of results (see Fig. 2B). They synchronized less consistently than controls with all stimuli [*t*_(33.2)_ = 4.34, *P* < 0.0001, *d* = 1.39]. Despite the adults outperformed children on this task [main effect of Age, *F*_(1,70)_ = 61.45, *P* < 0.00001, *η* ^2^_*partial*_ = 0.47], ADHD participants showed greater difficulties in tracking the beat of music than of tone sequences [Group x Stimulus interaction, *F*_(1,70)_ = 24.22, *P* < 0.00001, *η* ^2^_*partial*_ = 0.26; stimulus difference for ADHD, *t*_(41)_ = 6.22, *P* < 0.0001, *d* = 0.74; for controls, *t* < 1].

As tapping to the beat involved both a perceptual and a motor component, we tested whether poor motor control alone may account for the aforementioned group differences. Indeed, children and adults with ADHD showed higher motor variability than controls when tapping regularly without sound [vs. controls, *F*_(1,88)_ = 11.51, *P* = 0.001, *η* ^2^_*partial*_ = 0.12]. Summary statistics for children and adults in unpaced tapping are presented in Figure S3. Yet, when covarying for motor variability, ADHD children and adults still revealed lower synchronization performance than controls [main effect of Group, *F*_(1,67)_ = 31.06, *P* < 0.00001, *η* ^2^_*partial*_ = 0.32]. This difference was more apparent with music than with tones [Group x Stimulus interaction, *F*_(1,67)_ = 17.72, *P* < 0.0001, *η* ^2^_*partial*_ = 0.21] (see *SI Comparison of ADHD children and adults tapping performance* for ANCOVA results).

### Individual differences

**Figure 3:**
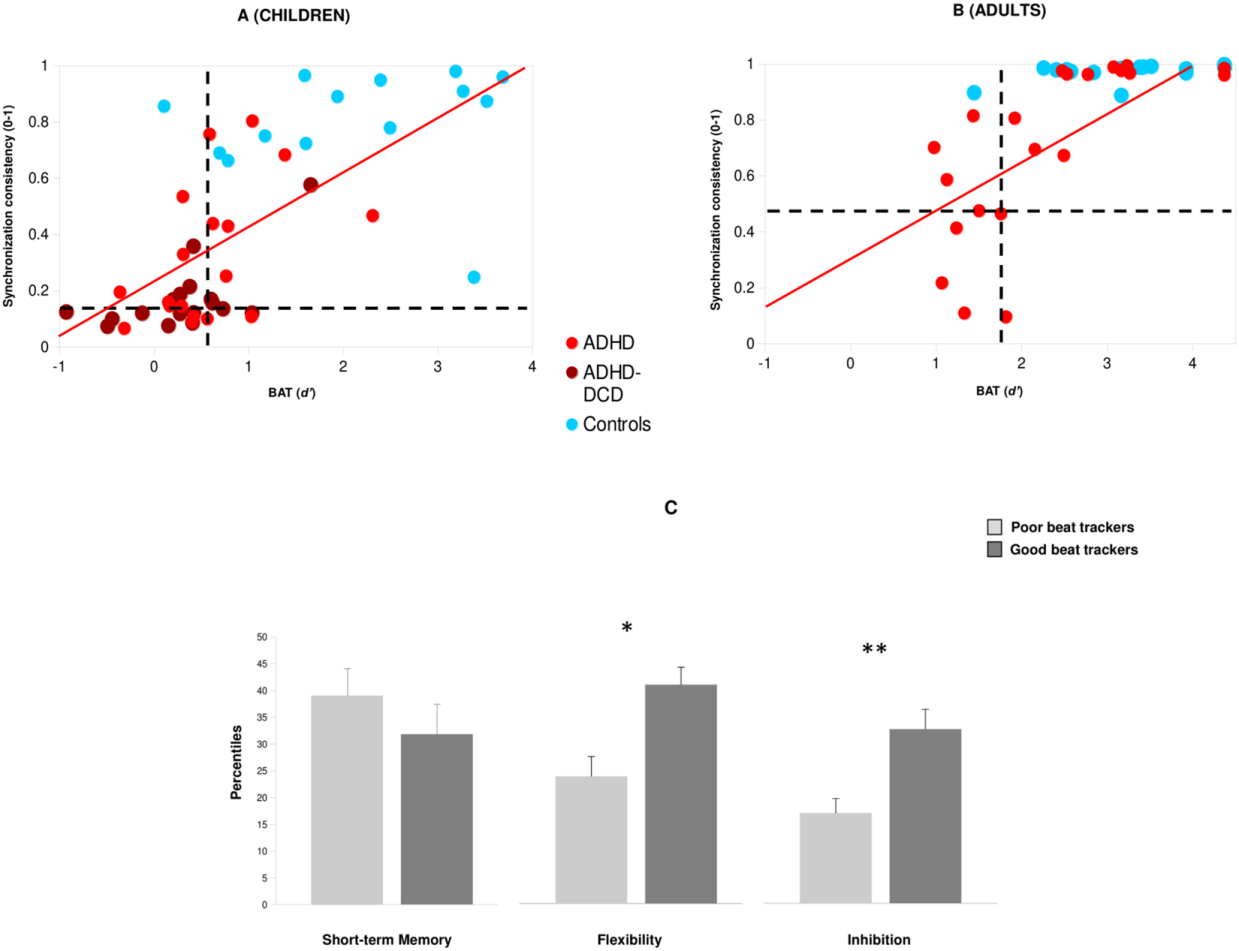
Individual performances in beat tracking as reflected by their results in the BAT (*d*’) and in paced tapping (synchronization consistency). Results from (**A**) children (ADHD, ADHD-DCD, and controls), and (**B**) adults (ADHD and controls). In both panels (A) and (B), dotted lines indicate the threshold used to identify participants as poor or good beat trackers, corresponding to −2 *SD* relative to the performance of the respective age-matched control group. (**C**) Cognitive functioning (short-term memory, flexibility, and inhibition) for children and adults with ADHD, divided into good and poor beat trackers. Error bars are SEM. **P < 0.01, *P < 0.05.

Despite group results provided compelling evidence of poor beat tracking in ADHD and ADHD-DCD, we observed important individual differences. They can be seen when plotting individual performances in the BAT task against synchronization consistency (Fig. 3A and B, for children and adults, respectively). Some ADHD participants (31.4% of children, and 38% of adults) still performed within the range of controls, while others showed very poor beat tracking skills. Scores in these two tasks are highly correlated [children, *rho* = 0.72, *P* < 0.0001; adults, *rho* = 0.80, *P* < 0.0001]; hence, they are likely to pinpoint the same beat tracking skills. Notably, because performance on the BAT does not rely on fine motor control, variability in beat tracking cannot be accounted for by differences in motor control.

Having shown variable beat tracking skills in ADHD, we tested whether this variability is associated with the severity of cognitive impairment, such as reduced flexibility, poor short-term memory, and poor inhibitory control^33–35^. To this aim, children and adults with ADHD were divided into good and poor “beat trackers”, based on the calculation of a *Beat Tracking Index* (BTI; see Materials and Methods). The BTI is a composite score based on normalized individual performances in the BAT and in paced tapping tasks of BAASTA, two tasks particularly sensitive to individual differences^11^. ADHD participants with a BTI score higher than −2 were classified as *good beat trackers*, and those with a score lower than −2, as *poor beat trackers*. Good beat trackers performed better than poor trackers on the Flexibility task and on the Inhibition task (see Fig. 3C) [*t*_(49.5)_ = 2.15, *P* < 0.05, *d* = 0.56, and *t*_(50.5)_ = 3.07, *P* < 0.01, *d* = 0.79, respectively]. In spite of the association between beat tracking and short-term memory sometimes reported^36^, good and poor beat trackers did not differ in that respect [*t* < 1]. In addition, children who were good and poor beat trackers did not differ in I.Q., and on measures of visual-selective, divided, and sustained attention, as well as on a general measure of auditory attention. Summary statistics for these measures are presented in Table S2.

## Discussion

Distortions in perceiving and producing single durations are a hallmark of ADHD^5, 37^. In this study we found that ADHD is also associated with rhythmic deficits (in keeping with^18^). Rhythmic skills were tested systematically with a vast array of perceptual and sensorimotor tasks, and with simple and complex rhythmic stimuli. Moreover, we demonstrate that children with ADHD, albeit they show sensitivity to subtle deviations from the beat in simple sound patterns, do not benefit from the rich rhythmic structure of music, when they perceive the beat or move to it. Extracting a beat based on music’s peculiar metrical structure (i.e., a system of embedded periodicities)^38^ appears as a particularly challenging task. This is visible in both perceptual and motor tasks, when children move to the beat. These difficulties are independent of children’s ability to discriminate single durations. Interestingly, poor beat tracking in musical contexts is not confined to childhood. Adults reveal the same pattern of results. Finally, we demonstrate that beat-tracking skills in children and adults are closely linked to inhibition and flexibility, two cognitive functions often impaired in ADHD.

Poor discrimination of durations in children with ADHD confirms impaired duration-based timing already documented in this condition^5, 37^. This deficit is associated with brain defects in the cerebellum and fronto-cerebellar pathways^39, 40^, usually linked to the processing of single durations^5, 9, 16^. The surprising finding that children still perceive durations in the context of a simple rhythm suggests that they may still benefit from a predictable temporal structure^11, 16^. However, the beneficial effect of a rhythmic context is not confirmed with music. Children and adults with ADHD struggle in tracking the beat of music. Because poor beat tracking with music is apparent in both perceptual and motor tasks we can conclude that this deficit is not merely the outcome of high motor variability typically found in ADHD^31^.

Music is particularly well-suited to test someone’s ability to track the beat. Unlike a metronome, the beat of music is not immediately provided by the temporal structure of a sequence of periodic sounds. With music, the beat has to be internally generated based on the complex pattern of durations characteristic of music sequences. This process has been found to engage a cortico-subcortical network, including in particular the basal ganglia (e.g., the putamen) and their connectivity with cortical areas (supplementary motor area and premotor cortex)^13, 25^. The possibility of a deficient system for internal generation of a beat is appealing, and consistent with the neuronal substrates of ADHD. There is compelling evidence that the aforementioned brain areas and their connectivity are affected in ADHD. This condition is associated with reduced blood flow in the putamen^23^ and lower functional connectivity between motor areas (primary and premotor) and the putamen as compared to controls^32^. In sum, poor beat tracking observed in ADHD may results from impaired internal generation of the beat, a function mostly linked to the basal ganglia.

Our findings are consistent with the results of a previous study by Serrallach and collaborators^18^, who showed difficulties in rhythm discrimination (but not in anisochrony detection with tones) in children with ADHD. Interestingly, in that study children with ADHD differed from children with Attention Deficit Disorder without Hyperactivity (ADD), who showed no impairment relative to controls. This suggests that hyperactivity may be an important factor to explain poor rhythm perception in ADHD, at least in children (but note, that we could not find differences in rhythm perception between adult subgroups with ADHD and ADD). However, there is a discrepancy with Serrallach *et al*.’s findings, in terms of duration discrimination. In their study, children with ADHD and ADD could compare two durations as healthy children did, while we found a deficit in this task in ADHD children (see also ref. 5). This difference may be linked to medication. The majority of the patients in Serrallach *et al*.’s study were under medication the day of the assessments, while this was not the case in the current study. It is likely that medication improved duration discrimination, as similar beneficial effects of methylphenidate were reported in previous studies^5, 41^.

Poor beat tracking in ADHD may also point to deficits in the way children and adults with this condition attend to events dynamically over time. This function, referred to as “dynamic attending”^42^ is supported by growing neurophysiological evidence (EEG) that in the healthy brain beat perception is driven by entrainment of neuronal populations which resonate at the beat frequency of an external rhythmic stimulus^43–45^. Oscillatory brain activity (e.g., in the beta band) is sensitive to the complexity of the rhythmic patterns^46, 47^ and can distinguish well-known metrical structures, such as marches and waltzes^47^. In a different context, this oscillatory model has proven valuable for explaining auditory disturbances and differences in cerebral activation found, notably, between dyslexic and non-dyslexic children^48^. Similarly, it may pave the way to linking the different brain substrates (cortical and subcortical), which have been associated with ADHD^49^. Thus, entrainment to an external rhythm as revealed by oscillatory brain behavior may be an additional indicator of beat-based deficits in ADHD. To date, EEG studies in ADHD, conducted in the absence of external stimulation (at rest) showed both increased power in the theta band and decreased power in the beta band^50^. Abnormal neural oscillations may pave the way to poor response to an external rhythmic stimulation in ADHD^44, 51, 52^. This interesting possibility remains purely speculative at this stage, though, and deserves further investigation.

As expected, tracking the beat in motor tasks was more difficult for children with ADHD and DCD than for children with ADHD. However, ADHD and ADHD-DCD children were similarly poor in tracking the beat in perceptual tasks. Thus, the source of the difference between the two conditions lies in motor processing, rather than generally beat tracking. This is in line with recent work showing specifically impaired sub-cortical/cortical connectivity in motor areas in DCD, relative to ADHD^32^. Nevertheless, ADHD and DCD share cerebral abnormalities, notably in the basal ganglia^53^, which may represent the neuronal underpinning of a core deficit with beat tracking found in both conditions. Because beat tracking skills in perceptual and motor tasks are highly correlated, performances in these tasks are likely to reflect a common beat-based mechanism which might go awry during development in ADHD^44^ and DCD.

To conclude, our findings demonstrate core deficits with tracking the beat of music in ADHD, with and without DCD, in children as well as in adults. These deficits may stem from the ability to generate the beat internally, a function engaging basal ganglia-cortical networks. Nevertheless, it has to be mentioned that beat tracking skills are quite heterogeneous in ADHD. The observed differences between ADHD participants and controls are found at a group level. Yet, it appeared that 35% of ADHD participants could track the beat as controls did, while the others showed impairment. This variability is not totally surprising, as ADHD is generally characterized by high heterogeneity in terms of cognitive functioning^34, 54^. Interestingly, we found that beat tracking skills covary with functions such as flexibility and inhibition, but not short-term memory. Poor beat trackers displayed lower flexibility and inhibition than controls. This finding is highly relevant from a clinical point of view. For example, it is still largely unknown whether medication (e.g., methylphenidate) affects beat tracking skills, together with the improvement of general cognitive functioning^5^. Beneficial effects of medication may indeed generalize to beat processing. There are indications that treatment of duration can be improved by methylphenidate^41^ while difficulties with rhythm perception persist in children with ADHD under medication^18, 55^. Thus, separable timing systems may be differentially affected by medication in ADHD^5, 41^.

Moreover, with other populations, there is evidence that variability in rhythmic skills before a dedicated training can be exploited to predict the success of the training^56^. For example, individuals with relatively spared rhythmic skills can benefit from a long-term training in which they move together with rhythmic sound (e.g., rhythmic auditory stimulation in patients with Parkinson’s disease)^56^. Owing to the link between rhythmic and cognitive functions^5, 8, 57^, rhythmic training, for example using perceptual or sensorimotor rhythmic exercises may hold some promise for remediation of cognitive disorders in ADHD, as observed with other neurodevelopmental disorders, like dyslexia and specific language impairments^58, 59^.

## Materials and Methods

### Participants

#### Children

Fifty-five children were recruited from the Montpellier area (France) to participate in the experiment. Forty-one were children with ADHD. Among them, 22 (2 females; 6 left-handed) were children with ADHD only, aged between 6.3 and 12 years (Mean = 8.7 years, *SD* = 1.5) and 19 (3 females; 4 left-handed) were children with ADHD and DCD, aged between 6.8 and 12.6 years (Mean = 8.8 years, *SD* = 1.7). Children in the ADHD group had received a diagnosis of ADHD based on DSM-5 criteria^1^ by a multidisciplinary team from the University Regional Hospital (CHRU) of Montpellier. Beyond the DSM-5 required criteria, and in order to ensure an accurate diagnosis of specific ADHD sub-types, the evaluation phase was complemented by semi-directed interviews with parents, and validated questionnaires had to be filled out by parents and teachers^60^. For each patient, an agreement on the final diagnosis was reached by a team of experts (three psychologists, one child psychiatrist, and one language therapist), and confirmed by a consultative advice from the referent child-psychiatrist.

Only children with ADHD of the combined type (with both hyperactive-impulsive and inattentive symptoms) were recruited. Children in the ADHD-DCD group received a diagnosis by the same hospital team. They performed below the 15^th^ percentile on the French version of the gold-standard M-ABC test^61, 62^, a developmental battery assessing motor disturbances. Children in both groups complained at the beginning of attentional, school or motor difficulties. They all scored above 70 on the I.Q. test^63, 64^. Other comorbid conditions such as dyslexia, neurological disorders, autistic spectrum disorders, sensory or physical disabilities were excluded. Finally, none of the children with ADHD (with or without DCD) was treated with methylphenidate the day of the experiment. This medication, typically administered to reduce inattention, hyperactivity and impulsive symptoms, has also beneficial effects on timing abilities^5, 41, 65^, thus potentially affecting measurement of timing skills. A third group of 14 healthy children (5 females; 1 left-handed) without ADHD, aged between 8.2 and 14.1 years (Mean = 9.4 years, *SD* = 1.6) were recruited for the Control group. Healthy participants did not show intellectual, cognitive, learning or motor disorders.

#### Adults

Thirty nine adults were recruited from the Montpellier area (France). Twenty-one (11 females; 1 left-handed) aged between 19.1 and 50.1 years (Mean = 31.4 years, *SD* = 10.5) formed the ADHD group. They received a diagnosis of ADHD based on DSM-5 criteria^1^, by a psychiatrist (RL) in a specialized outpatient clinic for adult ADHD. Eleven adults with ADHD were of the combined type (with both hyperactive-impulsive and inattentive symptoms), while 10 were of the inattentive type. None of the adults with ADHD was treated with methylphenidate during the week preceding the testing, and the day of the test. The other 18 participants (7 females; 3 left-handed), aged between 19 and 42.2 years (Mean = 32 years, *SD* = 7.2) formed the Control group. Healthy adults did not reveal signs of intellectual, cognitive, learning and motor disorders. The study was approved by the Institutional Review Board (n. 1610D) of the EuroMov research center. All experiments were performed in accordance with relevant guidelines and regulations. Informed consent was obtained from all children’s parents and from all adult participants.

### Measures of perceptual and sensorimotor timing skills

Perceptual and sensorimotor timing skills were assessed with the Battery for the Assessment of Auditory and Sensorimotor Timing Abilities^11^. BAASTA consists of a set of perceptual and motor tasks which proved sensitive to timing and beat tracking deficits in a variety of conditions (e.g., Parkinson’s disease, developmental stuttering, beat deafness, and tone deafness)^11, 19, 66–68^. In this study we selected five tasks from BAASTA for assessing beat tracking skills (3 perceptual tasks and 2 sensorimotor tasks), with simple and complex auditory material (sequences of tones and music). Perceptual tasks consisted in detecting deviations from the beat (Anisochrony detection), or in saying whether a superimposed metronome was aligned or not with a musical beat (Beat Alignment Test). Sensorimotor tasks involved finger tapping to the beat (Paced tapping). Two additional control tasks were performed to assess perception of single durations in the absence of beat tracking (Duration discrimination) and motor variability in the absence of an auditory stimulus (Unpaced tapping). Details for each task are provided below. Children were tested on all the tasks with a computer version of BAASTA. Auditory stimuli were delivered via headphones (Sennheiser HD201) and tapping data was acquired with a Roland SPD-6 MIDI tapping pad. Responses were provided verbally by the children, and entered by the Experimenter using the computer keyboard by pressing one of two keys corresponding to a “yes” or “no” response. “Yes” indicated the situation when the child detected a duration difference, the presence of an anisochrony, or that a metronome was misaligned with the musical beat. Adults were tested on the BAT and on the motor tasks with a tablet version of BAASTA (LG G Pad 8.0 model), while auditory stimuli were delivered via headphones (Sennheiser HD201). A response latency of 129 ms was measured for the MIDI tapping pad (precision of 1 ms) and was subtracted from the tapping data before further analysis. The measurements with the tablet interface had no latency, and precision below 1 ms.

The order of the tasks was fixed (Duration discrimination, Anisochrony detection with tones and music, BAT, for perceptual tasks; Unpaced tapping and Paced tapping to tones and music, for motor tasks).

### Perceptual tasks

#### Anisochrony detection with tones

With this task we tested participants’ ability to perceive a temporal irregularity (i.e., a time shift) in an isochronous sequence of tones - a metronome^69, 70^. Participants listened to sequences of five tones (tone duration = 150 ms; frequency = 1047 Hz). While some sequences (20% of all trials) were isochronous and presented with a constant inter-onset interval (IOI = 600 ms), others contained a deviation from isochrony. This corresponds to a time shift of the fourth tone that occurred earlier than expected based on the previous sounds. The amount of the time shift, up to 30% of the IOI, was changed adaptively depending on the participants’ response. This was implemented via a Maximum Likelihood Procedure^70^ using the MATLAB MLP toolbox^71^. The task was to judge whether the sequence was “regular” or “irregular”. Participants performed 3 blocks of 16 trials each.

#### Anisochrony detection with music

As done in the previous task, participants detected a time shift. However, here the time shift corresponded to a deviant beat in a short musical excerpt^11, 69^. In each trial, a computer-generated musical excerpt from Bach’s “Badinerie” (orchestral suite for flute BWV 1067) was played with a piano timbre at a tempo of 100 beats/min (IOI = 600 ms; beat = quarter note). The excerpt was played in a regular version (with isochronous beats) or in an irregular version with a time shift introduced at the onset of the fifth beat. The magnitude of the time shift, up to 30% relative to the IOI was controlled by the MLP algorithm. The task was to tell whether the rhythm was “regular” or “irregular”. As before, there were 3 blocks of 16 trials each.

#### Beat Alignment Test (BAT)

With this task we tested listeners’ ability to detect deviations from the beat. The task is an adapted version of the BAT^72^. Participants listened to four musical excerpts with a salient beat: two from Bach’s « Badinerie » and two from Rossini’s « William Tell Overture ». Each excerpt included 20 beats (beat = quarter note). After the seventh beat, a sequence of isochronous tones with a triangle timbre (a metronome) was superimposed onto the music. The tones were either aligned or not to the musical beat. When unaligned, the tones occurred earlier or later than the beat by 33% of the quarter note duration, or the interval between the tones was increased or decreased by 10% of the quarter note duration. The stimuli were presented at three different tempos (IOIs = 450, 600, and 750 ms), for a total of 72 stimuli. Stimuli were presented in randomized order. Participants judged whether the metronome was aligned or not to the beat of music.

#### Duration discrimination (control task)

In this test we assessed whether participants could perceive single durations, in the absence of an underlying beat. They were presented with two tones (frequency = 1 kHz, interval between tones = 600 ms). The first tone (standard) lasted 600 ms, and the second, between 600 and 1000 ms. Participants judged if the second tone lasted longer than the first. The duration of the second tone was controlled by the MLP algorithm. There were 3 blocks, each including 16 trials.

### Motor tasks

#### Paced tapping to tones

We tested whether participants could track the beat by asking them to tap with their finger to sequences of isochronously presented sounds. Each sequence included 60 piano tones (tone frequency = 1319 Hz). The tones were presented at three tempos (IOIs = 450, 600, and 750 ms).

All stimuli were repeated twice.

#### Paced tapping to music

The same task as above was carried out with musical stimuli. Participants were asked to tap with their finger to the beat of two excerpts taken from Bach’s « Badinerie » and from Rossini’s « William Tell Overture » (quarter note IOI = 600 ms). Each excerpt included 64 beats and was repeated twice.

#### Unpaced tapping (control task)

We tested participants’ motor variability in a tapping task in which they did not have to track the beat. Their task was to produce regular fingers taps with their dominant hand at a comfortable rate for 60 seconds^73^. The tasks were carried out twice.

### Data analysis

*Perceptual tasks*: for Duration discrimination and Anisochrony detection tasks, the perceptual thresholds obtained in the three blocks were averaged and expressed in percentage of the stimulus IOI (Weber ratio). Blocks with more than 30% of false alarms were discarded^11^. In the BAT, the sensitivity index (*d’*) was calculated, as an unbiased measure of detection performance, based on the number of Hits (when unaligned tones were correctly detected) and False alarms (when lack of alignement was incorrectly reported). *d’* is the difference between the z-transform of Hits rate and False Alarm rate.

*Motor tasks*: for both Paced and Unpaced tapping tasks, the first ten taps were discarded. For the Unpaced tapping task, the mean *tapping rate* (the mean inter-tap interval, ITI) and *motor variability* were calculated. Motor variability was the coefficient of variation of the ITI (CV of the ITI), namely the ratio of the *SD* of the ITIs over the mean ITI. For Paced tapping tasks, synchronization of the taps to the stimulus beat was calculated with circular statistics^11, 73, 74^. Individual taps were expressed as angles on a polar scale from 0 to 360 deg., considering that the full circle corresponds to the inter-beat interval. Angles were treated as unit vectors and used to calculate the mean resultant vector *R* ^73–75^. The length of vector *R*, ranging from 0 to 1, indicates *synchronization consistency* (i.e., the reciprocal of variability)^11, 69, 74–76^. A value of 1 means that all the taps occured exactly at the same time interval before or after the pacing stimulus (maximum consistency); 0 means absence of synchronization (the taps are randomly distributed between the beats). Before statistical analyses, synchronization consistency was submitted to a logit transformation^11, 68^.

*Beat Tracking Index* (*BTI*): this is a global measure of beat tracking skills computed by considering the performance of both the BAT and Paced tapping tasks from BAASTA. The source data to compute the BTI was the overall sensitivity index (*d*’) obtained from the BAT, and synchronization consistency in paced tapping averaged across stimuli (tones and music) and tempos. *Z*-scores for values of *d’* and synchronization consistency were independently calculated for children and adults [*z*-score = (value − Mean_controls_)/*SD*_controls_], while taking mean and *SD* of their respective control groups. The BTI was calculated by averaging the *z*-scores obtained for the BAT and paced tapping. Participants with BTI scores lower than −2 (i.e., with a performance lower than 2 *SD* relative to their matched control group) were treated as “poor beat trackers”, while the others were considered as “good beat trackers”.

### Neuropsychological measures

Children and adults with ADHD were submitted to neuropsychological tests for assessing their cognitive functioning (general intelligence, memory, attention and executive functions). Different tasks were administered to children and adults. For comparison, raw scores obtained in tests of attentional and executive functions were converted into percentiles.

#### Children

The neuropsychological tests included measures from the WISC-IV intelligence scale, from which we took into consideration the composite total I.Q. score, and the score from the digit span task, as a measure of short-term memory^63, 64^. In the latter task, children repeated numbers in the same order as presented aloud by the examiner. For backward digit span, the children repeated numbers in the reverse order of that presented by the examiner. I.Q. scores were converted into composite scores (Mean = 100, *SD* = 15). Moreover, the digit span score was converted into percentiles based on normative data.

Six attentional and executive functions were assessed with the Test of Everyday Attention for Children^77^. They included selective visual attention, auditory attention, flexibility, divided attention, inhibition, and sustained attention. The tests of these functions were administered in the following order.

*Selective visual attention*: in this timed task, pairs of four different types of spaceship drawings were presented. Most pairs included two different spaceship drawings. Children had to find the pairs of identical spaceship drawings (target items) as quickly as possible. Twenty target items were distributed among 108 pairs with different drawings (i.e., distractors). The performance score takes into account the number of good pairs of drawings circled and the time spent to do it.

*Auditory attention*: children listened silently to 10 series of tones. Each series included between 5 and 16 tones. Children had to report how many sounds they heard at the end of each series. The score is the number of good responses.

*Flexibility*: in this attention visual-switching task presented in a booklet the participant had to count the number of “creatures” (i.e., green little monsters) visible all along their burrow. Arrows interspersed among the creatures pointed either upwards or downwards. The children were instructed to begin counting the creatures one by one and to change the direction of their counting when the sense of the next arrow was downwards, until the last creature was presented. The final score took into account the number of good responses and the time spent to complete the task.

*Divided attention*: children performed simultaneously two tasks, namely a visual-search task and an auditory counting task. The individual tasks are similar to the aforementioned auditory and visual selective attention tasks. The final score took into account the number of good responses both on the visual task and on the auditory task, and the total time spent to complete the test. The test took end when the children thought they found all visual stimuli they could.

*Inhibition*: in this Go/No go task, children pointed with a felt-tip pen to a series of squares drawn on a sheet as they heard short sequences of tones (from 4 to 16 tones). They were asked to stop pointing when a different timbre occurred. The rate of presentation of the tones increased progressively. The score is the number of good responses.

*Sustained attention*: this is a test of vigilance in the auditory modality. The children monitored a stream of digits presented at a rate of one/2 sec. They were instructed to detect a particular target sequence (two consecutive “5”), and to report the digit occurring immediately before the sequence. Forty targets were presented over 16 min. The score is the number of good responses.

#### Adults

Adults were administered the computerized Test of Attentional Performance^78^, for the evaluation of three attentional and executive domains: inhibition, flexibility, and short-term memory.

The tests were administered in the following order.

*Inhibition*: in this Go/No go task, an up-right (“+”) cross and a diagonal (“×”) cross were presented in an alternating sequence on the screen. Participants were asked to press a button as quickly as possible only when the diagonal cross (i.e., the target) appeared. The score corresponds to the number of wrong responses (i.e., when a participant pressed the button when an up-right cross appeared on the screen).

*Flexibility*: in this set-shifting task, in each trial a letter and a digit were simultaneously presented on the right or left side of a computer screen. Participants were asked to press the left or right button on the keyboard as fast as possible depending on the side of appearance of the letter, then of the digit. The final score takes into account the number of good responses and the reaction time for each item.

*Short-term memory*: this task examines the control of information flow and the updating of information in short-term memory. A sequence of digits was presented to the participants on the computer screen. Participants pressed a button when the digit visible on the screen was the same as the penultimate one. The performance score is the number of omissions made by the participant.

### Statistical analysis

Independent samples *t*-tests and mixed-design ANOVAs were used to compare ADHD participants (with and without DCD) and controls on BAASTA tests. In ANOVAs, Stimulus was the within-subjects factor (tones vs. music) and Group (ADHD vs. controls) the between-subjects factor. Further ANOVAs tested the differences between children and adults, by taking Age (children vs. adults) as an additional between-subject factor. When differences between children with ADHD and ADHD-DCD did not reach significance, data were pooled. Post-hoc paired and independent samples *t*-tests with Bonferroni correction were performed to define observed effects. Spearman correlations instead of Pearson were performed between variables whenever the data were not normally distributed. Statistics were computed using R software^79^.

## Electronic supplementary material

Supplementary information
