# Supplementary material for: Children and adults with Attention-Deficit/Hyperactivity Disorder cannot move to the beat
Source: Sci Rep. 2017 Sep 14;7:11550. doi: 10.1038/s41598-017-11295-w (PMC5599521; doi:10.1038/s41598-017-11295-w)
Supplement: Supplementary file 1 — Supplementary information [file 41598_2017_11295_MOESM1_ESM.pdf]

SUPPLEMENTARY INFORMATION

Children and adults with Attention-Deficit/Hyperactivity Disorder cannot move to the beat

Frédéric Puyjarinet[1], Valentin Bégel[1,2], Régis Lopez[3,4], Delphine Dellacherie[5,6], & Simone Dalla Bella[1,7,8,9*]

[1] EuroMov Laboratory
University of Montpellier
700 Av. du Pic Saint Loup
34090 Montpellier, France

[2] NaturalPad, SAS
700 Av. du Pic Saint Loup
34090 Montpellier, France

[3] National reference center for narcolepsy and idiopathic hypersomnia, specialized in adult ADHD, Gui-de-Chauliac University Hospital
80 Av. Augustin Fliche
34295 Montpellier, France

[4] Inserm Unit U1061, La Colombière University Hospital
39 Av. Charles Flahault
34093 Montpellier, France

[5] Department of Psychology, PSITEC-EA 4072 Laboratory, University of Lille
Domaine Universitaire « Pont de bois »
59653 Villeneuve d'Ascq, France

[6] University Hospital of Lille, Department of Pediatric Neurology
2 Av. Oscar Lambret
59037 Lille, France

[7] International Laboratory for Brain, Music and Sound Research (BRAMS)
1430 Boulevard du Mont-Royal
Montreal, QC H2V 2J2, Canada

[8] Institut Universitaire de France
1 Rue Descartes
75231 Paris, France

[9] Department of Cognitive Psychology, WSFiZ in Warsaw
Ul. Pawia 55
01-030, Warsaw, Poland

*Corresponding Author: Simone Dalla Bella (PhD), simone.dalla-bella@umontpellier.fr
+33 (0)434 432 649

Timing and beat perception

Effects of age in children with ADHD and controls

There were no statistically-significant differences between ADHD children and the control group in terms of age [$t_{(16.1)} = 1.05$, $P = 0.31$, $d = 0.36$]. Additionally, we tested potential correlations between age and cognitive measures (short-term memory, flexibility, and inhibition) as well as perceptual and sensorimotor rhythmic skills. Correlations did not reach significance for cognitive measures [average $r = 0.06$, $P = 0.75$], and for beat perception tasks [average $r = -0.05$, $P = 0.71$]. However, performance in paced tapping slightly improved with age [$r = 0.29$, $P < 0.05$].

Differences between ADHD and ADHD-DCD children performance

Mean performance in timing and beat perception tasks for children with ADHD only and with ADHD-DCD are reported in Table S1. Discrepancies in the samples size for each task are due to the fact that in some cases the thresholds could not be reliably computed. The thresholds in the Duration discrimination and Anisochrony detection tasks were obtained by averaging the values obtained in the three blocks, expressed in percentage of IOI (Weber ratio). Blocks with more than 30 % of false alarms (FAs, when a difference for a catch trial is reported) were removed. Moreover, blocks leading to aberrant threshold estimations due to persistent local minima in the maximum-likelihood procedure, or due to a lack of convergence of the estimation function at the end of a block, were rejected. In the latter case, the convergence of the estimated threshold was assessed by calculating the slope of local threshold values across the last eight trials of a block. Lack of convergence was indicated by a slope exceeding 10 % relative to the mean threshold of the preceding trials (for details, see Dalla Bella et al., 2017). In addition, one participant did not carry out the BAT task, and data could not be correctly recorded for 4 participants in this task due to technical issues.

As can be seen in Table S1, children with ADHD did not differ significantly from children with ADHD-DCD on all the perceptual tasks. Their thresholds did not differ significantly in the Duration discrimination task [$t_{(28.7)} = 1.16$, $P = 0.26$, $d = 0.40$]. To compare the performance of the two sub-groups in the Anisochrony detection tasks, thresholds were submitted to a 2 (Group) x 2 (Stimulus) mixed-design ANOVA, taking Group (ADHD vs. ADHD-DCD children) as the between subject factor and Stimulus (tones vs. music) as the within-subject factor. Neither the main effects of Group and Stimulus, nor their interaction was significant [$Fs < 1$]. Finally, even if a tendency was apparent for children with ADHD-DCD to perform worse than children with ADHD on the BAT, this difference did not reach significance [$t_{(31.8)} = 1.71$, $P = 0.098$, $d = 0.57$].

Table S1. Results obtained by ADHD and ADHD-DCD children on all perceptual tasks of BAASTA. Statistical comparisons of the two subgroups are reported.

Task		ADHD		ADHD-DCD		*t* value (*df*)	*P*
		Mean (*SE*)	*n*	Mean (*SE*)	*n*		
Duration discrimination (threshold)		36.98 (*2.79*)	19	31.84 (*3.45*)	15	1.16 (*28.70*)	0.26
Anisochrony detection (threshold)	With tones	17.23 (*1.35*)	20	17.09 (*1.79*)	18	0.12 (*35.00*)	0.9
	With music	15.35 (*2.16*)	14	16.98 (*2.64*)	12	0.48 (*24.00*)	0.63
BAT (*d'*)		0.73 (*0.20*)	19	0.31 (*0.14*)	17	1.71 (*31.80*)	0.09

Comparison of ADHD children and adults' BAT performance

The performance of children (pooled data for ADHD and ADHD-DCD sub-groups) and adults with ADHD on the BAT at the three tempos (450, 600, and 750-ms Inter-Beat-Interval, IBI) is summarized in Figure S1. The discriminability index (*d'*) was entered in a 2 (Age) x 2 (Group) x 3 (Tempo) mixed-design ANOVA. Age (children vs. adults) and Group (ADHD vs. controls) were the between-subject factors, and Tempo (450 vs. 600 vs. 750-ms IBI), the within-subject factor.

Children overall had more difficulties than adults to detect misaligned beats [main effect of Age, $F_{(1,85)} = 51.11$, $P < 0.00001$, $\eta^2_{partial} = 0.38$]. The performance differed as a function of Tempo [main effect of Tempo; $F_{(2,170)} = 7.41$, $P < 0.001$, $\eta^2_{partial} = 0.07$]. All participants showed worse performance with music at the fast tempo (450-ms IBI) than at the average tempo [vs. 600-ms IBI, $t_{(88)} = 3.17$, $P < 0.01$, $d = 0.18$], or at the slow tempo [vs. 750-ms IBI, $t_{(88)} = 3.78$, $P < 0.001$, $d = 0.22$]. Interactions between the aforementioned factors did not reach significance.

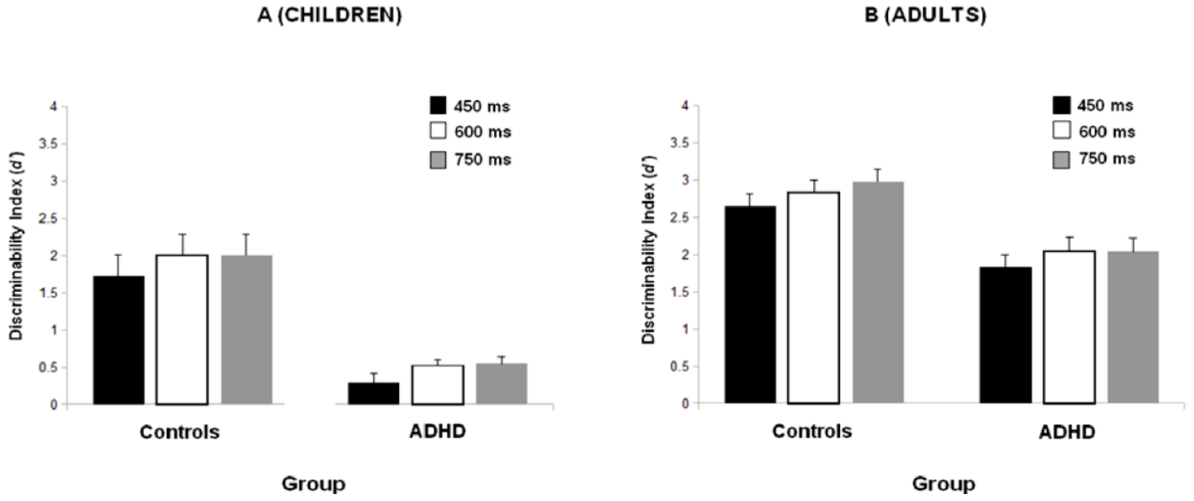

Figure S1. Performances of children and adults on the BAT (i.e., *d'* at 450-ms, 600-ms, and 750-ms IBI). Error bars are SEM.

Tapping to the beat

Children performance when they tapped to the beat of tone sequences (a metronome) is summarized in Figure S2. To assess whether their performance on this task varied as a function of stimulus rate (or beat tempo), synchronization consistency (following logit transformation) was entered in a 3 (Group) x 3 (Tempo) mixed-design ANOVA. Group (ADHD vs. ADHD-DCD vs. controls) was the between-subject factor, and Tempo (450 vs 600 vs 750-ms Inter-Stimulus-Interval, IOI), the within-subject factor. A main effect of Group was found [$F_{(2,51)} = 17.60$, $P < 0.00001$, $\eta^2_{partial} = 0.41$], confirming the results of a previous ANOVA taking the average performance across tempos. Neither the effect of Tempo [$F_{(2,102)} = 2.15$, $P = 0.12$, $\eta^2_{partial} = 0.04$] nor the Group x Tempo interaction [$F_{(4,102)} = 1.15$, $P = 0.34$, $\eta^2_{partial} = 0.04$] reached significance.

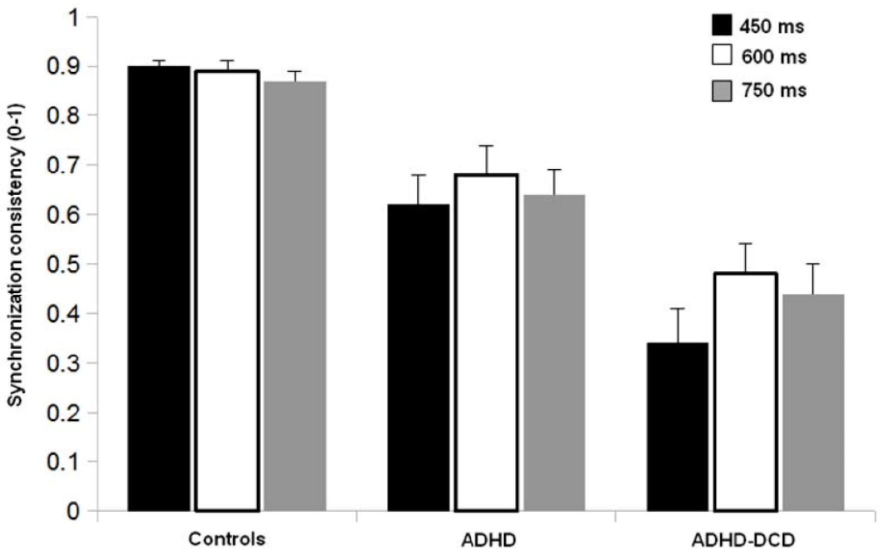

Figure S2. Performances obtained by children (Controls, children with ADHD, and children with ADHD-DCD) in the paced tapping task with tones sequences at the three tempos. Error bars indicate SEM.

Comparison of ADHD children and adults tapping performance

The performance of children and adults in the unpaced tapping task was analyzed first. The mean of the inter-tap intervals (ITIs) and the coefficient of variation of the ITI (i.e., *SD* of the ITI / mean ITI) were calculated. The mean ITI indicates the *tapping rate*, and the coefficient of variation of the ITI a measure of *motor variability*. Mean tapping rate and motor variability for children and adults are reported in Figure S3. Tapping rate and motor variability were submitted to separate 2 (Age) x 2 (Group) ANOVAs. Both Age (children vs. adults) and Group (ADHD vs. controls) were between-subject factors. No differences were found in terms of tapping rate between children and adults [main effect of Age, $F_{(1,88)} = 1.02$, $P = 0.32$, $\eta^2_{partial} = 0.01$], and between ADHD and controls [mean effect of Group, $F < 1$]. The Age x Group interaction was not significant [$F < 1$]. However, differences were observed in terms of motor variability. Children were more variable than adults [main effect of Age, $F_{(1,88)} = 12.29$, $P < 0.001$, $\eta^2_{partial} = 0.12$] and participants with ADHD were more variable than controls [main effect of Group, $F_{(1,88)} = 11.51$, $P = 0.001$, $\eta^2_{partial} = 0.12$]. The Age x Group interaction just failed to reach

significance [$F_{(1,88)}$ = 3.70, P = 0.06, $\eta^2_{partial}$ = 0.04], suggesting that the difference between ADHD participants and controls may be more important for children than for adults. Note that this difference may be biased by the presence of children with DCD among children with ADHD. Indeed, when the same analysis was repeated considering only children with ADHD without DCD, the difference between ADHD participants and controls was no more significant [$F_{(1,69)}$ = 1.54, P = 0.22, $\eta^2_{partial}$ = 0.02].

Differences between adults and children in the paced tapping tasks were tested by comparing adults with ADHD to children with ADHD (without DCD) and their respective controls. This ensured unbiased comparison of children and adults, given that a difference was found between ADHD and ADHD-DCD children in the tapping tasks. Synchronization consistency (after logit transformation) was submitted to a 2 (Age) x 2 (Group) x 2 (Stimulus) mixed-design ANOVA. Age (children vs. adults) and Group (ADHD participants vs. controls) were the between-subject factors, and Stimulus (tones vs. music), the within-subject factor. The analysis showed main effects of Age [$F_{(1,70)}$ = 61.45, P < 0.00001, $\eta^2_{partial}$ = 0.47], Group [$F_{(1,70)}$ = 50.15, P < 0.00001, $\eta^2_{partial}$ = 0.42], and Stimulus [$F_{(1,70)}$ = 25.99, P < 0.00001, $\eta^2_{partial}$ = 0.27]. Moreover, there were two significant interactions between Age and Stimulus [$F_{(1,70)}$ = 5.31, P < 0.05, $\eta^2_{partial}$ = 0.07], and between Group and Stimulus [$F_{(1,70)}$ = 24.22, P < 0.00001, $\eta^2_{partial}$ = 0.26]. The triple interaction did not reach significance. To control for the aforementioned group differences in motor variability, an ANCOVA was run in which the same factors as above were considered and motor variability, obtained from the unpaced tapping task, was taken as a covariate. The main effects of Age, Group, and Stimulus remained highly significant as a result of the ANCOVA (Ps < 0.01). The interaction between Group and Stimulus was still highly significant [$F_{(1,67)}$ = 17.72, P < 0.0001, $\eta^2_{partial}$ = 0.21]. This was not the case for the Age x Stimulus interaction [$F_{(1,67)}$ = 3.34, P = 0.07, $\eta^2_{partial}$ = 0.05].

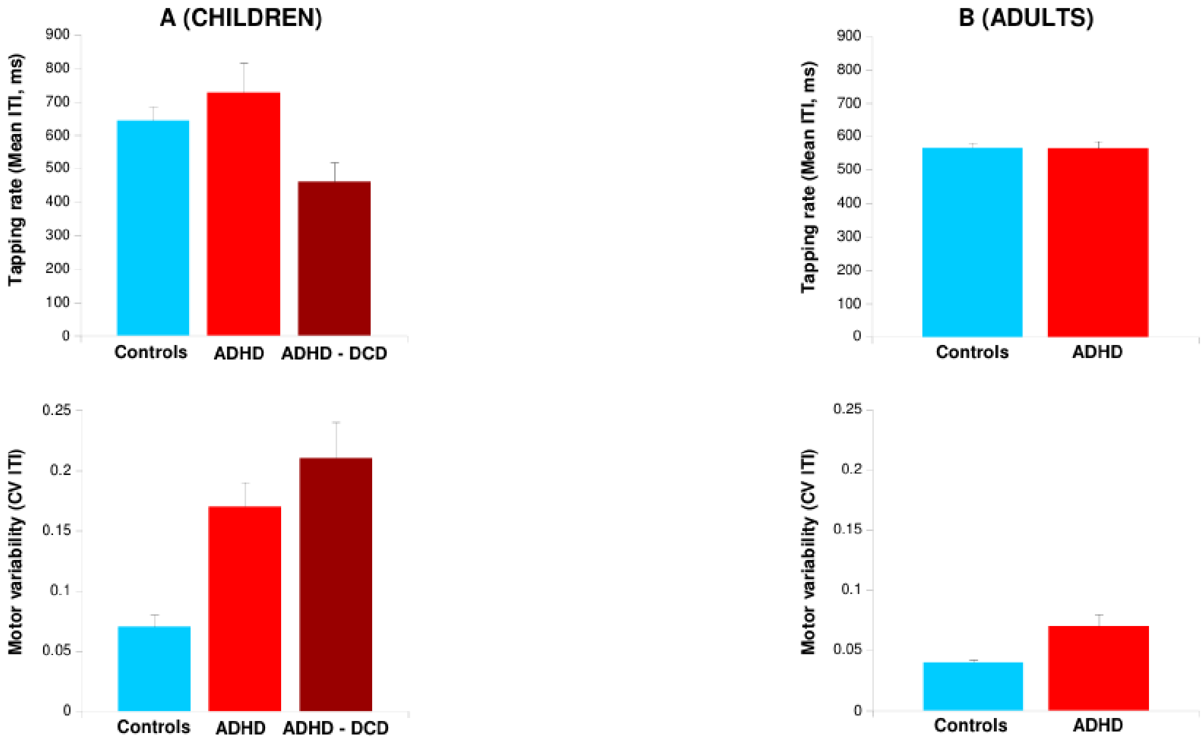

Figure S3. Tapping rate (mean ITI) and motor variability (CV of the ITIs) for children (A) and adults (B) obtained in the unpaced tapping task.

Individual differences

Further tests were conducted to tests whether children with ADHD, divided into good and poor beat trackers, differed on measures of I.Q., selective attention, divided attention, sustained attention, auditory attention, short-term memory, inhibition, and flexibility. These measures are reported in Table S2. The discrepancies of the sample sizes for the different tasks result from the inability of some children to complete all the tasks until the end of the session due to evident fatigue or restless behavior. No significant differences between good and poor beat trackers were found on all tasks, except for inhibition.

Table S2. Average measures in cognitive tests for ADHD children identified as good and poor beat trackers.

Measure	Poor beat trackers		Good beat trackers		t value (*df*)	*P*
	Mean (*SE*)	*n*	Mean (*SE*)	*n*		
I.Q.	92.56 (*3.56*)	18	98.91 (*3.98*)	11	1.19 (*23.54*)	0.25
Short-term memory	35.64 (*1.75*)	17	45.40 (*3.27*)	10	0.77 (*17.60*)	0.22
Selective attention	30.42 (*6.88*)	24	40.00 (*7.58*)	17	0.94 (*36.16*)	0.36
Divided attention	27.92 (*5.29*)	24	27.19 (*7.04*)	16	0.09 (*30.40*)	0.93
Sustained attention	31.90 (*6.31*)	21	27,00 (*6.30*)	16	0.55 (*34.31*)	0.59
Auditory attention	31.83 (*7.40*)	24	32.80 (*8.47*)	15	0.09 (*32.13*)	0.93

Potential influence of gender and hyperactivity on beat-tracking measures

To test whether differences between females and males affected the results in the different groups, factorial ANOVAs were run on the different measures of beat tracking in adults (total *d*-prime for the BAT, paced tapping with music and metronome, and unpaced tapping) by taking both Group and Gender as between-subject factors. The analyses showed no significant effects of Gender (for BAT, paced tapping, and unpaced tapping, *F*s < 1), nor interactions between Gender and Group (for BAT and unpaced tapping, *F*s < 1; paced tapping, [$F_{(1,90)} = 3.23$, $P = 0.08$, $\eta^2_{partial} = 0.03$]).

In addition, to assess the potential influence of hyperactivity in the ADHD adult group, a comparison of adults with combined type with those showing a predominant inattentive type was run on the same beat-tracking measures. None of these difference reached significance, thus showing that the presence of hyperactivity did not significantly influence our results (for BAT, paced tapping, and unpaced tapping, *t*s < 1).

Reference

Dalla Bella, S., Farrugia, N., Benoit, C.E., Bégel, V., Verga, L., Harding, E., Kotz, S.A. BAASTA: Battery for the Assessment of Auditory Sensorimotor and Timing Abilities. *Behav Res Methods*, **49**(3), 1128-1145 (2017).
